# BAC transgenic mice provide evidence that p53 expression is highly regulated *in vivo*

**DOI:** 10.1038/cddis.2015.224

**Published:** 2015-09-17

**Authors:** L Chen, G X Zhang, Y Zhou, C X Zhang, Y Y Xie, C Xiang, X Y He, Q Zhang, G Liu

**Affiliations:** 1 MOE Key Laboratory of Model Animal for Disease Study, Model Animal Research Center, Nanjing Biomedical Research Institute, Nanjing University, 12 Xuefu Road, Pukou District, Nanjing, Jiangsu 210061, China; 2 Jiangxi University of Traditional Chinese Medicine, 18 Yunwan Road, Nanchang, Jiangxi 330004, China; 3 Institute of Biophysics, Chinese Academy of Sciences, 15 Datun Road, Chaoyang District, Beijing 100101, China

## Abstract

p53 is an important tumor suppressor and stress response mediator. Proper control of p53 level and activity is tightly associated with its function. Posttranslational modifications and the interactions with Mdm2 and Mdm4 are major mechanisms controlling p53 activity and stability. As p53 protein is short-lived and hardly detectable in unstressed situations, less is known on its basal level expression and the corresponding controlling mechanisms *in vivo*. In addition, it also remains obscure how p53 expression might contribute to its functional regulation. In this study, we established bacterial artificial chromosome transgenic E.coli *β*-galactosidase Z gene reporter mice to monitor *p53* expression in mouse tissues and identify important regulatory elements critical for the expression *in vivo*. We revealed preferentially high level of *p53* reporter expressions in the proliferating, but not the differentiated compartments of the majority of tissues during development and tissue homeostasis. In addition, tumors as well as regenerating tissues in the *p53* reporter mice also expressed high level of *β*-gal. Furthermore, both the enhancer box sequence (CANNTG) in the *p53* promoter and the 3′ terminal untranslated region element were critical in mediating the high-level expression of the reporter. We also provided evidence that cellular myelocytomatosis oncogene was a critical player regulating *p53* mRNA expression in proliferating cells and tissues. Finally, we found robust p53 activation preferentially in the proliferating compartment of mouse tissues upon DNA damage and the proliferating cells exhibited an enhanced p53 response as compared with cells in a quiescent state. Together, these results suggested a highly regulated expression pattern of *p53* in the proliferating compartment controlled by both transcriptional and posttranscriptional mechanisms, and such regulated p53 expression may impose functional significance upon stress by setting up a precautionary mode in defense of cellular transformation and tumorigenesis.

p53, encoded by *p53* (also termed as *TP53* gene or transformation related protein 53 gene (*Trp53*)), is one of the most important tumor suppressors. Constant degradation in normal embryos or tissues mediated by the E3-ligase activity of Mdm2 renders p53 protein difficult to be detected,^1^ whereas Mdm4 mainly inhibits the transcriptional activity of p53.^2^ Repression of these two inhibitors by stress stimuli is the major posttranslational regulatory mechanism controlling p53 activity and stability.^3^ Once activated, p53 is able to mediate a plethora of responses including inhibition of cellular proliferation important for tumor suppression.

A number of studies demonstrated the importance of *p53* gene dosage and expression level in affecting its activity and the cellular responses. Apoptosis increased with p53 levels in cultured cells.^4^ In mice, additional 1–2 copies of wild type *p53* did not impact mouse development, growth and senescence, but significantly enhanced the sensitivity to *γ*-irradiation and strengthened the resistance to tumorigenesis.^5^ On the other hand, *p53* haploinsufficiency was observed in a variety of situations including tumorigenesis and stress responses. Both individuals harboring a *p53* germline mutation in the Li–Fraumeni families and *p53* heterozygous mice exhibited increased incidence of tumors, some of which apparently did not lose the wild-type allele. In some instances, p53 heterozygosity was able to rescue the lethal phenotype caused by deletion of *Mdm2*^6^ or *Mdm4,*^7^ again demonstrating a dosage dependent phenotype. In relevance to human breast cancer, methylations in CG rich region of *p53* promoter impaired its transcription and protein levels,^8^ and impaired expression of the transcriptional factor *HOXA5* also decreased *p53* mRNA level in the tumor tissues.^9^ All these implied the importance of *p53* expression and its regulatory mechanisms for stress responses and tumor suppression.

Early studies indicated a ubiquitous tissue expression pattern of *p53* mRNA,^10^ consistent with p53's role as a tumor suppressor of tissues from different origins. However, the studies on distribution of *p53* mRNA in sub-tissue compartments have been scarce and scattered. *In situ* hybridization found high-level *p53* mRNA expression during embryonic development^11^ and a striking *p53* mRNA expression pattern in postnatal rat brain, with intensive signals in subventricular zone, rostral migratory stream and external granular layer (EGL), where new neurons were produced.^12^ In NIH3T3 cells, *p53* mRNA level fluctuated with cell cycle progression.^13, 14^ F9 cells, an embryonic carcinoma stem cell line, expressed high levels of *p53* mRNA, which dropped to low levels after induction of differentiation.^15, 16^ Meanwhile, many efforts focused on identifying and analyzing the *cis*-elements and transacting factors important for *p53* expression. Cellular myelocytomatosis oncogene (c-Myc),^17^ NF-kappaB,^18, 19^ E2F1,^20^ C/EBP beta,^21^ EGR-1,^22^ and Ets-1/2^23^ were among the many transcription factors shown to regulate *p53* promoter activity. Other studies also addressed the posttranscriptional regulatory mechanisms for p53 expression. *Cis*-elements including AU rich element, CPE (cytoplasmic polyadenylation element) and micro RNA-binding sites were found present in *p53* 3′ terminal untranslated region (3′UTR) and regulated reporter *p53* mRNA stability and/or translational efficiency in either a positive or negative manner. A recent study also found that ectopic human *p53* 5′UTR and 3′UTR in H1299 cells could bind each other through paired bases and strengthened translational efficiency of the reporter transcript upon DNA damage.^24^ However, the *in vivo* relevance, contribution and concerted nature of these regulations on p53 expression remain incompletely understood.

Proliferation is the fundamental cellular process closely linked to development, homeostasis and cancer. There are evidence suggesting that fast proliferating cells are more sensitive to stresses and p53 activation. Conventional radiotherapy and chemotherapy often lead to severe side effects in cancer patients mainly affecting fast renewing tissues. Intriguingly, loss of *Mdm2* in a *p53* hypomorphic background resulted in p53 stabilization preferentially in the proliferating compartments of the postnatal mice, leaving important clues about p53 basal expression.^6^ More works are needed to decipher or distinguish the underlying mechanisms influencing the p53 regulatory disparities.

In this study, we established bacterial artificial chromosome (BAC) transgenic reporter mice to model basal *p53* expression in intact tissues during development, homeostasis, regeneration and tumorigenesis. We also evaluated the role of *p53* promoter and 3′UTR elements in governing p53 expression patterns. Our results clearly demonstrated a preferential high-level expression of p53 reporter in the proliferating compartments of multiple tissues as dictated by the enhancer box sequence (E-box) and 3′UTR regulatory elements. Importantly, we provided evidence that the regulation of basal level expression was closely correlated with the robustness of p53 response under stress conditions.

## Results

### Generation of BAC transgenic reporter mice in monitoring *p53* expression

**Figure 1:**
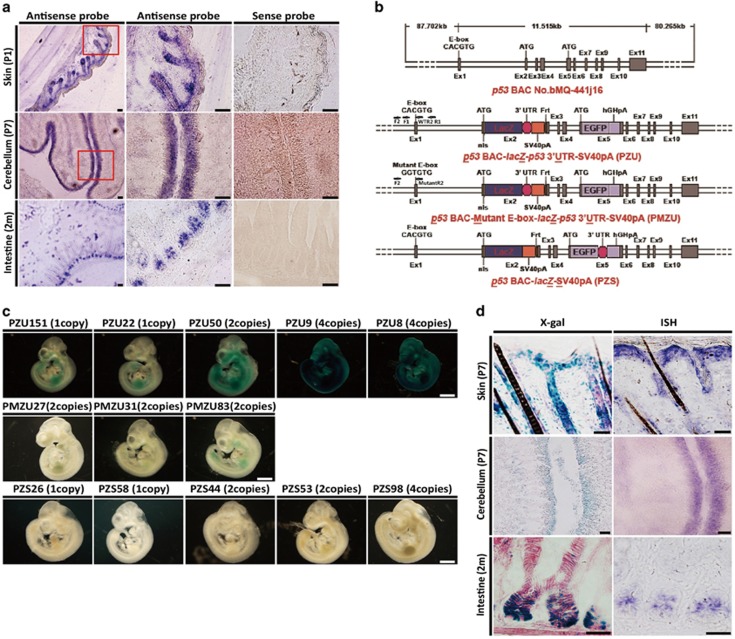
Generation and validation of BAC transgenic reporter mice to monitor *p53* expression. (**a**) *In situ* hybridization of *p53* mRNA using sense and antisense probes on postnatal day 1 mouse skin, P7 cerebellum and 2-month-old mouse intestine. Boxed areas in left panel were shown in the middle panel. Crypts in intestine were also shown in the middle bottom panel. Scale bar=100 *μ*m. (**b**) Schematic representations of the *p53* gene locus in the BAC and its modifications with the insertions of *lacZ* reporter in the ATG (exon 2) of full length p53 and *EGFP* reporter in the ATG (exon 5) of an isoform (Δ157) together with alterations in the regulatory elements in three BAC transgenic lines. ChIP primers used for detecting wild-type E-box-binding in tissues and cells (F1, R1 or F2, and WTR2); ChIP primers for detecting mutant E-box-binding in tissues and cells (F2, MutantR2). (**c**) *β*-gal staining of E10.5 mouse embryos from *PZU*, *PMZU* and *PZS* transgenic reporter lines with 1–4 BAC copies. Embryos were whole-mount stained in 1 mg/ml X-gal staining solution with gentle shaking for 24 h at 4 °C. Scale bar=400 *μ*m. (**d**) A comparison of *p53* reporter expression in p7 skin, p7 cerebellum and 2-month-old mouse small intestine by *β*-gal staining with *p*53 mRNA *in situ* hybridization. Scale bar=50 *μ*m

To probe *p53* mRNA expression pattern in mouse tissues, we performed *in situ* hybridization (ISH) in skins of postnatal day 1 mice, cerebellum of P7 mice and small intestine of mice at 2 months of age. *p53* mRNA was at high levels in the hair follicle and basal layer of the skin, EGL of the cerebellum and intestinal crypts (Figure 1a), which are actively proliferating; in contrast, *p53* mRNAs were at much lower levels in supra basal layer of the skin, internal granular layer (IGL) of the cerebellum and villus of the small intestine (Figure 1a), which belong to the differentiated compartments.

To study *p53* gene expression pattern more systematically, and to determine the regulatory mechanisms critical for basal *p53* expression *in vivo*, we established BAC transgenic mice in which a E.coli *β*-galactosidase Z gene (*LacZ*) reporter gene (with nuclear localization signal) followed by the *p53* 3′UTR and simian vacuolating virus 40 polyadenylation sequences (SV40pA) was inserted immediately after the start codon (ATG) in the exon 2 of the BAC *p53* gene locus. In addition, we disrupted a reported p53^*Δ*157^ isoform with start codon in *p53* exon 5^25, 26^ with an enhanced green fluorescence protein (EGFP)-human growth hormone polyadenylation sequences cassette in the same transgenic construct. No obvious EGFP expression was detected, and thus was not further investigated in this study. This transgenic model was designated as *p53*^***PZU***^ (or *PZU*) to monitor basal *p53* expression by *β*-galactosidase (*β*-gal) activity *in vivo* (Figure 1b). To study the transcriptional control of *p53* expression, we focused on a previously identified conserved E-box-binding motif close to the transcriptional start site in *p53* promoter.^17^ Chromatin immunoprecipitation (ChIP) analysis demonstrated a direct binding of c-Myc on this E-box sequence in proliferating cells but not in quiescent cells (Supplementary Figure 1). To address the possible regulatory role of the E-box in the *p53* promoter, *p53*^*PMZU*^ (or *PMZU*) transgenic mice were similarly established as *p53*^***PZU***^ except for a mutated E-box in the BAC *p53* promoter from *CAC*GTG to *GGT*GTG (Figure 1b). To assess the importance of *p53* 3′UTR in the regulation of basal *p53* expression, we also established *p53*^*PZS*^(or *PZS*) BAC transgenic mice in which the inserted *LacZ* reporter gene was followed only by the SV40pA to monitor the expression of *p53* without its own 3′UTR sequence (Figure 1b). With X-gal incubation, all five lines of *PZU* embryos exhibited clearly visible *β*-gal staining, the level of which correlated with their corresponding transgenic BAC copy numbers (Figure 1c). Notably, all the embryos from transgenic lines of *PMZU* and *PZS*, including the high copy number lines, exhibited severely diminished *β*-gal staining (Figure 1c). Comparisons of *LacZ* expressions in the skin, cerebellum and small intestine of the *PZU* mice with the *p53* mRNA ISH revealed highly consistent patterns (Figure 1d), validating our transgenic approach in monitoring endogenous *p53* mRNA expression.

### High level of *p53-LacZ* reporter expression in the proliferating compartments of mouse tissues

**Figure 2:**
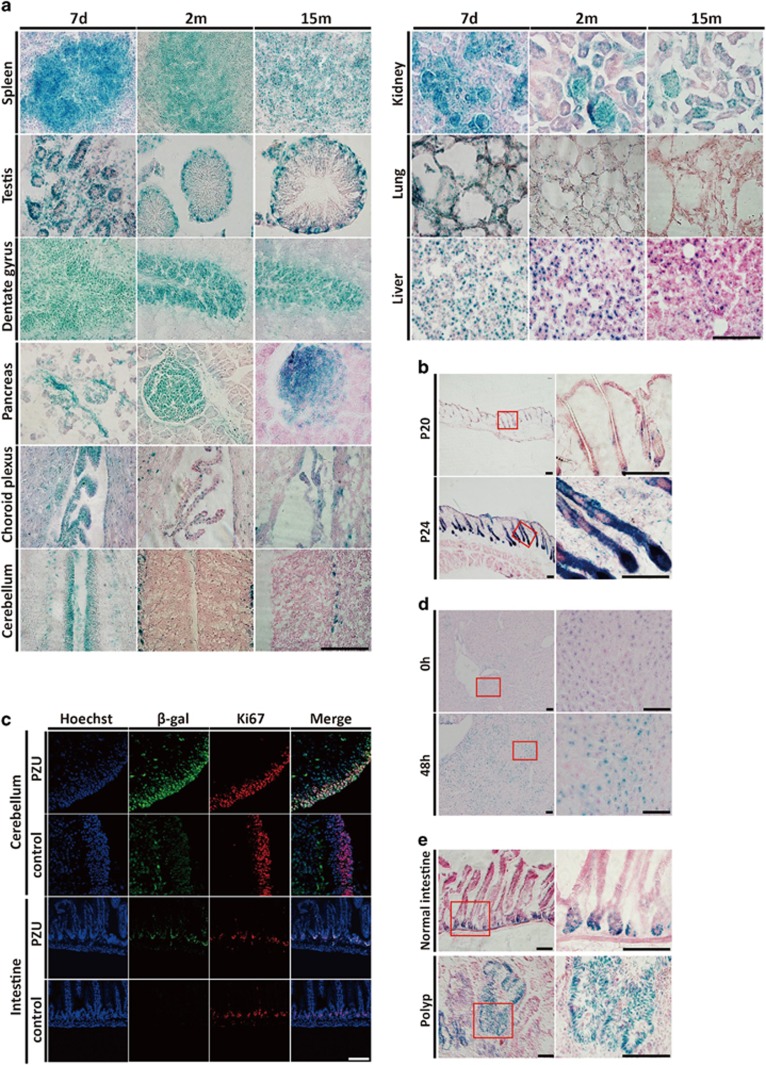
Preferential *p53* reporter expression in the proliferating compartments during mouse postnatal development, tissue homeostasis, regeneration and tumorigenesis. (**a**) *β*-gal staining of tissues including spleen, testis, dentate gyrus, pancreas, choroid plexus, cerebellum, kidney, lung and liver at P7, 2 months and 15 months of age respectively in *PZU* mice. Frozen slices of tissues were stained in 1 mg/ml X-gal staining solution at 37 °C for 10 h, except for 3 h in testis. Scale bar=200 *μ*m. (**b**) *β*-gal staining of skins at the anagen (p20) or telogen (p24) phase of the hair cycle in *PZU* mice. Frozen slices of skins were stained in 1 mg/ml X-gal staining solution at 37 °C for 10 h. Boxed areas were shown in the right panel. Scale bar=200 *μ*m. (**c**) Double immunofluorescence of *β*-gal and Ki67 in p8 cerebellum and adult small intestine of *PZU* mice. Scale bar=200 *μ*m. (**d**) *β*-gal staining of liver before and 48 h after hepatotectomy. Frozen slices of livers were stained in 1 mg/ml X-gal staining solution at 37 °C for 7 h in *PZU* mice. Boxed areas were shown in the right panel. Scale bar=200*μ*m. (**e**) *β*-gal staining of small intestine in *PZU* mice and polyps in *PZU*; *Apc*^*min*^ mice

To reveal *p53* expression pattern and levels in mouse tissues, we performed X-gal staining in tissues of *PZU* mice at postnatal day 7–8, 2–3 months and 13–15 months of age, respectively. Intense *β*-gal staining was observed in multiple tissue compartments including germinal center of spleen, spermatogenous cells of seminiferous tubule in testis, dentate gyrus (DG) of hippocampus, islet of postnatal and adult pancreas, ependymocytes of choroid plexus, EGL of the postnatal cerebellum, renal tubule and glomerulus, and pulmonary alveoli (Figure 2a). In contrast, *β*-gal was expressed at low or undetectable levels in the differentiated cells toward the center of the seminiferous tubule, mature neurons outside the DG region in adult hippocampus, acinous cells in adult pancreas and the IGL of cerebellum (Figure 2a), which were all non-proliferating in nature. Meanwhile, direct comparisons in the same tissues revealed that *β*-gal-staining intensities decreased when mice aged, especially in spleen, choroid plexus, kidney and lung (Figure 2a). Further, the whole mount X-gal staining on de-skinned mice also demonstrated attenuated *β*-gal expression in mouse muscle and skeleton with age (Supplementary Figure 2a). As few exceptions, the non-proliferating hepatocytes in the adult liver and purkinje cells of the cerebellum also demonstrated detectable levels of *β*-gal staining in the *PZU* mice (Figures 2a and d).

The preferential expressions of *p53-LacZ* reporter in proliferating compartment of tissues were further evaluated in the hair follicles of mouse skin which undergo step-wise growing and quiescent cycles. There were abundant strong *β*-gal-staining positive cells at the anagen stage of the hair cycle at P20, but only few at the quiescent telogen stage at P24 (Figure 2b). To study the link of proliferating or quiescent state with *p53-LacZ* expression, we performed X-gal staining in *PZU* MEFs either at proliferating or quiescent state. Upon withdraw of serum, proliferating MEFs gradually moved to a quiescent state. In parallel, *PZU* MEFs with high *β*-gal-staining intensity dropped from 46.2 to 2.6%, whereas cells with low-level *β*-gal increased from 18.7 to 68.3% (Supplementary Figure 2b). Next, we performed double immunofluorescence of ki-67 and *β*-gal in cerebellum and small intestine of the p7 *PZU* mice. *β*-gal positive cells were largely overlapped with those of Ki67 positive proliferating cells, establishing a direct link between high *p53-LacZ* expression and cellular proliferating state in multiple tissue compartments (Figure 2c).

Similar to the self-renewal of homeostatic tissues, tissue repair and regeneration also features cellular proliferation. *β*-gal expression was examined in the liver of *PZU* mice under hepatotectomy. In spite of the detectable basal level of *β*-gal staining, an enhanced *β*-gal expression was observed on the proliferation peak at 48 h (Figure 2d) as demonstrated by Ki67 staining (Supplementary Figure 2c), paralleling the level of endogenous *p53* mRNA expression (Supplementary Figure 2d).

Tumorigenesis is marked by uncontrolled proliferation. To study *p53* reporter expression during tumorigenesis, we crossed the *PZU* mice with *Apc^min^* mice with a mutation in the *Apc* gene and studied *β*-gal expression in the adenomas developed in the small intestine of the *PZU; Apc*^*min/+*^ mice. Most cells in these tumors expressed high levels of *β*-gal (Figure 2e), which paralleled with Ki67 immunostaining (Supplementary Figure 2e), whereas high levels of *β*-gal was only confined to the crypts in normal intestine (Figure 2e).

Collectively, high level of *β*-gal expression was specifically observed in the proliferating cell compartments in a variety of homeostatic or pathological contexts in the *PZU* mice, suggesting *p53* expression is highly regulated in the proliferating compartments.

### *p53* reporter expression is controlled by both the *p53* promoter E-box element and 3′UTR

**Figure 3:**
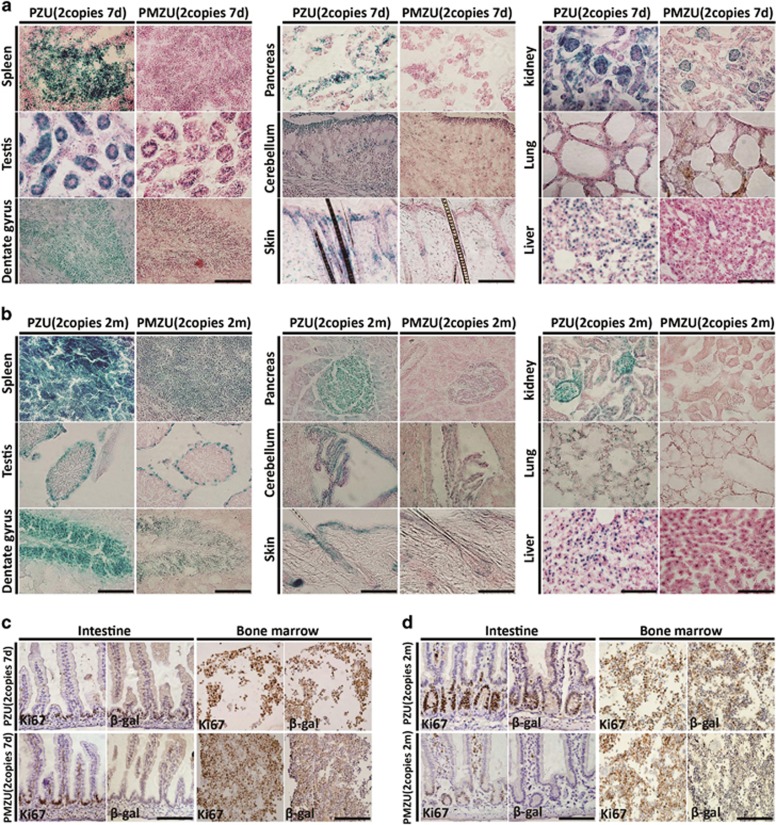
The E-box element in *p53* promoter was critical in driving high level of *p53* reporter expression in proliferating cell compartments. (**a** and **b**) *β*-gal staining of tissues at P7 (**a**) and 2 months (**b**) of age in *PZU* and *PMZU* mice. Scale bar=200 *μ*m. (**c** and **d**) IHC of Ki67 and *β*-gal on intestine and bone marrow from P7 (**c**) and 2-month-old (**d**) *PZU* and *PMZU* mice. Scale bar=200 *μ*m

**Figure 4:**
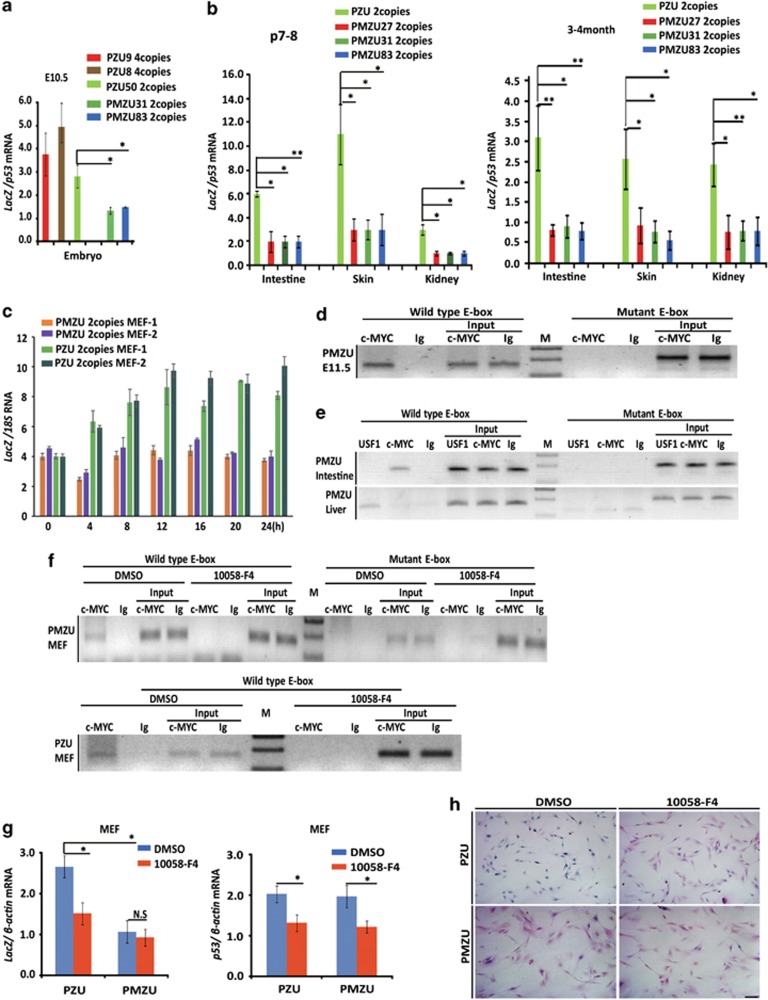
Disrupting c-Myc binding on the conserved E-box in *p53* promoter downregulated *p53* expression. (**a**) RT-PCR results of *LacZ* mRNA expression as normalized to *p53* mRNA in embryos at 10.5 day from *PZU* and *PMZU* mice. (**b**) RT-PCR results of *LacZ* mRNA expression as normalized to *p53* mRNA in small intestine, skin and kidney from *PZU* and *PMZU* mice. (**c**) RT-PCR results of *lacZ* mRNA expression normalized to 18 s RNA in a time course upon serum stimulation in MEFs from *PZU* and *PMZU* mice with the same BAC copy number. (**d**) ChIP analysis on c-Myc binding on the endogenous *p53* promoter (wild-type E-box) or *p53* promoter in transgenic BAC (mutant E-box) in the E11.5 embryo of the *PMZU* mice. (**e**) ChIP analysis on c-Myc and USF binding on the endogenous *p53* promoter (wild type E-box) or *p53* promoter in transgenic BAC (mutant E-box) in small intestine and liver of 2-month-old *PMZU* mice. (**f**) ChIP analysis on c-Myc binding on the endogenous p53 promoter (wild-type E-box) or p53 promoter in transgenic BAC (mutant E-box) in the MEFs from the *PMZU* mice under the treatment of Myc inhibitor 10058-F4 for 6 h (upper panel) and ChIP analysis on c-Myc binding on the p53 promoter (wild-type E-box) in the MEFs from the *PZU* mice under the treatment of Myc inhibitor 10058-F4 for 6 h (lower panel). (**g**)RT-PCR results of *LacZ* and *p53* mRNA expressions normalized to *β*-actin in MEFs from *PZU* and *PMZU* mice under the treatment of 10058-F4 for 8 h. (**h**) *β*-gal staining of MEFs from the *PZU* and *PMZU* mice under the treatment of 10058-F4 for 10 h. MEFs were stained in 1 mg/ml X-gal staining solution at 37 °C for 10 h. Scale bar=200 *μ*m. Values are means±S.E.M.s. *P<0.05; **P<0.01; ***P<0.001 (*t*-test)

With the E-box-mutated *PMZU* mice and 3′UTR-deleted *PZS* mice, we addressed the respective roles of the conserved E-box and the *p53* 3′UTR sequence on the enhanced reporter expression in the proliferating cells during development and homeostasis. Significantly diminished *β*-gal staining was observed in the *PMZU* embryos as compared with the *PZU* embryos with the same BAC copy number (Figure 1c). At both P7-8 and 2–3 months of age, *β*-gal staining was all reduced in the *PMZU* mice as compared with the *PZU* mice in a panel of tissues examined, including spleen, testis, hippocampus, pancreas, choroid plexus, postnatal cerebellum, skin, lung and liver (Figures 3a and b). Immunohistochemistry (IHC) with *β*-gal antibody also revealed decreased *β*-gal expressions in the small intestine and bone marrow of the *PMZU* mice, whereas IHC of Ki67 remained the same between the *PMZU* and *PZU* mice (Figures 3c and d). Consistently, in the *PMZU* mice, the *L*acZ mRNA level normalized to that of endogenous *p53* also dropped by 50–73% in embryos, postnatal and adult tissues as compared with those of the *PZU* mice (Figures 4a and b). These results indicated an important role of the E-box on *p53-LacZ* reporter expression *in vivo*.

Cell cycle exit, reentry and progression can be manipulated in cell culture systems. *p53* mRNA was elevated 8 h after serum stimulation and reached its peak at 16 h, paralleling cell cycle progression^13, 14^ (Supplementary Figure 3). *LacZ* mRNA from *PZU* MEFs also exhibited a similar elevation upon serum stimulation, whereas *LacZ* mRNA level from the *PMZU* mice remained unchanged (Figure 4c), again suggesting a role of E-box in coordinating cell cycle progression and *p53* expression. c-Myc is a major Leucine Zipper-bHLH transcription factor important in promoting proliferation and cell cycle progression in many types of cells. To study whether the reduction of *β*-gal expression in proliferating tissues of *PMZU* mice is due to the loss of binding of c-Myc or other transcriptional factors to the mutant E-box, ChIP assay was performed in developing embryos and adult intestine, as well as the non-proliferating adult liver in the *PMZU* mice containing both the wild-type E-box from the endogenous *p53* locus and the mutant E-box from the transgenic reporter. As expected, c-Myc could bind wild-type E-box in the endogenous *p53* promoter, but not the mutant E-box in transgenic *p53* promoter in the proliferating tissues of the *PMZU* mice (Figures 4d and e). However, little c-Myc binding was observed on endogenous *p53* promoter in liver (Figure 4e). In contrast, upstream stimulatory factor-1 (USF-1), another bHLH transcription factor, could bind the E-box in endogenous *p53* promoter but not the mutant E-box in liver of the *PMZU* mice (Figure 4e). Notably, no USF-1 binding to endogenous *p53* promoter in small intestine was observed (Figure 4e). These results suggested a differential binding of transcription factors to the *p53* E-box element in tissues. To reveal the functional significance of c-Myc binding in regulating *p53* expression, specific Myc–Max dimerization inhibitor 10058-F4 was used to treat proliferating MEFs. 10058-F4 significantly impaired c-Myc binding on the wild-type E-box in both *PMZU* and *PZU* MEFs (Figure 4f) and decreased both *LacZ* mRNA expression and *β*-gal staining in *PZU* MEFs (Figures 4g and h). Furthermore, although 10058-F4 treatment also resulted in decreased endogenous *p53* mRNA levels in both *PZU* and *PMZU* MEFs, it did not further influence the levels of *LacZ* mRNA and *β*-gal staining in *PMZU* MEFs (Figures 4g and h). Together, these results indicated a significant role of c-Myc in upregulating *p53* reporter expression through the E-box element, suggesting that c-Myc may be one of the major transcriptional factors driving high level of *p53* mRNA expression in proliferating cells.

**Figure 5:**
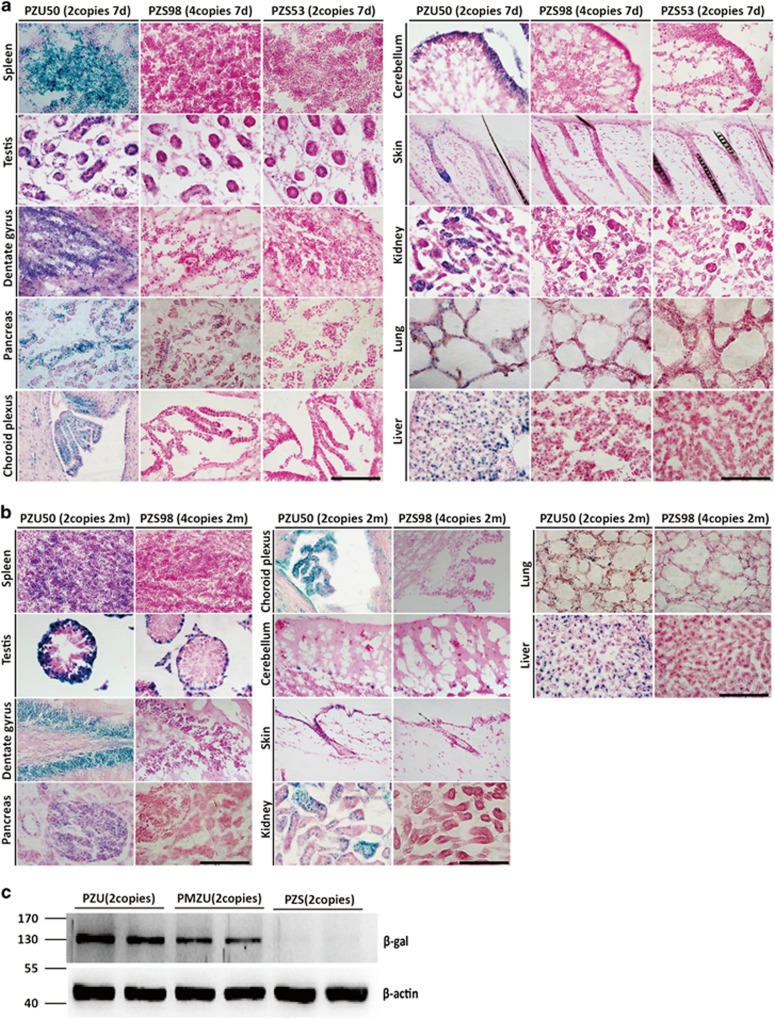
p53 3′UTR was essential in supporting high level of p53 reporter expression. (**a**) *β*-gal staining of multiple tissues including spleen, testis, dentate gyrus, pancreas, choroid plexus, cerebellum, skin, kidney, lung and liver in P7 *PZU* and *PZS* mice. Slices of frozen tissues were stained in 1 mg/ml X-gal staining solution at 37 °C for 10 h, except for 3 h for testis. Scale bar=200 *μ*m. (**b**) *β*-gal staining of multiple tissues including spleen, testis, dentate gyrus, pancreas, choroid plexus, cerebellum, skin, kidney, lung and liver in 2-month-old *PZU* and *PZS* mice. Slices of frozen tissues were stained in 1 mg/ml X-gal staining solution at 37 °C for 10 h, except for 3 h for testis. Scale bar=200 *μ*m. (**c**)Western blot analysis of *β*-gal expression in MEFs from *PZU*, *PMZU* and *PZS* mice with the two copies of transgenic BAC

Compared with *PZU* mice, *PZS* mice lacking *p53* 3′UTR exhibited no or very low-level *β*-gal expression visible only in limb bud of the E10.5 embryos with 1–4 BAC copies (Figure 1c). In addition, no or very weak *β*-gal staining was detected in multiple tissue compartments of the *PZS* mice with 2 or 4 BAC copies at both P7 (Figure 5a) and 2 months of age (Figure 5b). Only the *PZS* mice with 4 BAC copies exhibited weak *β*-gal staining in the spermatogenous cells of somniferous tubules in testis and dentate gyrus of hippocampus (Figures 5a and b). These results indicated an essential role of *p53* 3′UTR in supporting high level of *β*-gal expressions in the proliferating tissue compartments. Interestingly, in both embryos and a variety of adult tissues examined, *LacZ* mRNA levels of the *PZS* mice were not significantly different from those of the *PZU* mice (Supplementary Figure 4), indicating that *p53* 3′UTR does not significantly influence *LacZ* mRNA levels.

Finally, we directly compared the *β*-gal protein level in MEFs from the *PZU*, *PMZU* and *PZS* mice with two BAC copies. Western blot analysis showed that *β*-gal level dropped by >50% in *PMZU* MEF, and was almost undetectable in *PZS* MEFs (Figure 5c), consistent with the *β*-gal staining results in multiple tissues. These results further supported that *p53* E-box and 3′UTR were critical in sustaining high-level expression of the reporter gene.

### A functional link between cellular proliferating state and p53 response

**Figure 6:**
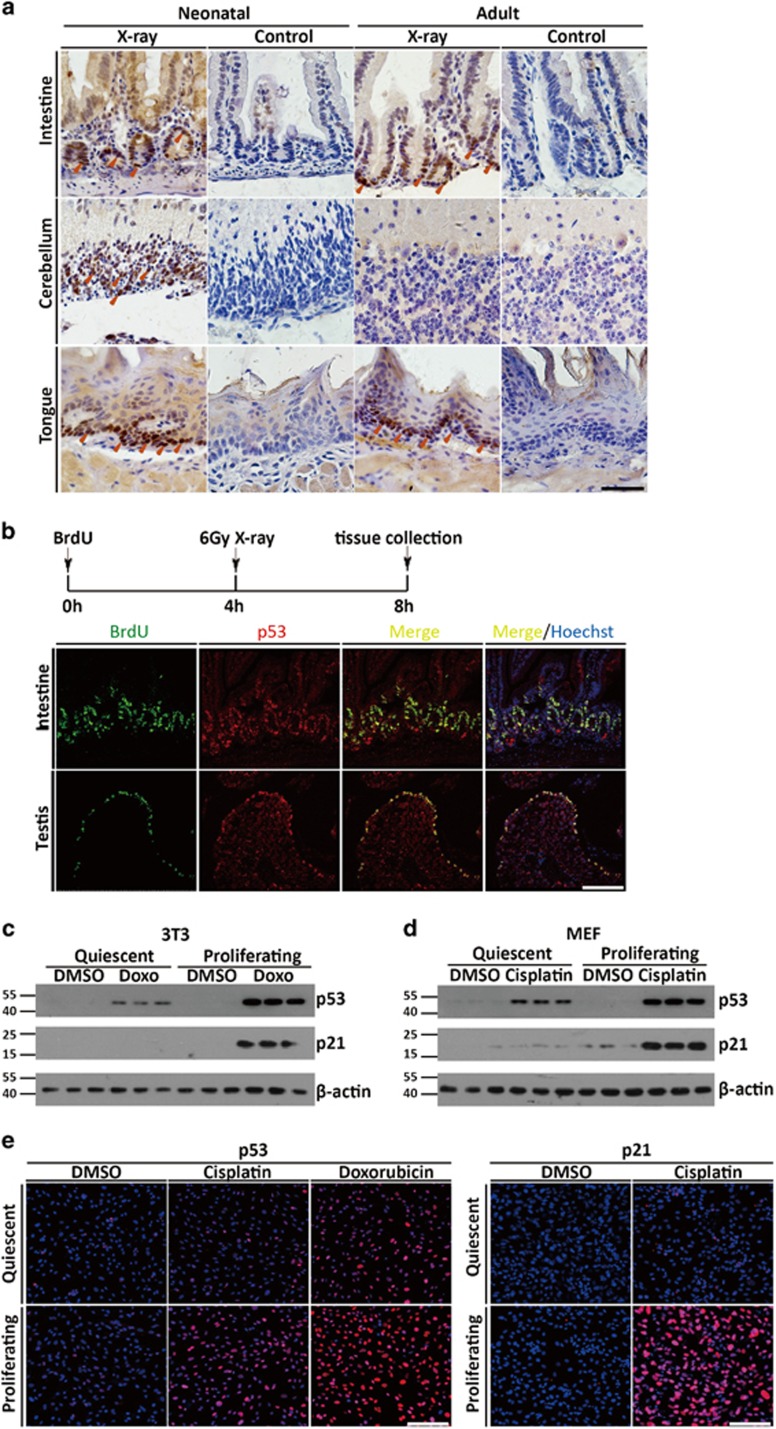
A direct link between cellular proliferation and p53-mediated stress response. (**a**) IHC of p53 in small intestine, cerebellum and tongue of p7 and 2-month-old C57BL/6J control mice or mice treated with X-irradiation for 4 h. Arrows depicted positive stained cells. Scale bar=200 *μ*m. (**b**) Double immunostaining of p53 and BrdU in intestine and testis of 2-month-old C57BL/6J mice treated with X-irradiation for 4 h. (**c**) Western blot analysis of p53 and p21 expression on quiescent and proliferating 3T3 cells treated with Doxorubicin. (**d**) Western blot analysis of p53 and p21 expression on quiescent and proliferating MEFs treated with Cisplatin. (**e**) Immunofluorescence of p53 or p21 in quiescent and proliferating MEFs treated with Cisplatin or Doxorubicin. Scale bar=200 *μ*m

Our findings that *p53* reporter expression was preferentially upregulated in proliferating cells suggested the possibility of a more robust p53 response upon stresses in proliferating cells compared with quiescent and differentiated cells. To directly explore the possible regulatory difference of p53 *in vivo*, B6 mice was either untreated or treated with 6 Gray of X-irradiation and immunostained for p53 protein in multiple tissue compartments. Consistent with previous results, p53 protein was undetectable in normal tissues from untreated mice (Figure 6a). In contrast, upon irradiation, p53 positive cells were readily detected in EGL of cerebellum in the postnatal mice, and in the basal cells of tongue and intestine crypt of both postnatal and adult mice (Figure 6a). In contrast, there was weak or no p53 staining in the more differentiated cell compartments of these tissues, such as IGL of the adult cerebellum, villi of small intestines and differentiated cells in the tongue epithelium (Figure 6a). Double staining with p53 and 5-Bromo-2-Deoxy Uridine (Brdu) antibodies further revealed that p53-positive-staining largely overlapped with that of Brdu in proliferating compartment of fast turn-over tissues including small intestine and testis (Figure 6b). Therefore, p53 protein was preferentially expressed in the proliferating tissue compartment upon induction, essentially recapitulating its gene expression pattern.

To directly analyze p53 functional differences in cells at different proliferating state, quiescent and proliferating cells were treated with either Doxorubicin or Cisplatin. p53 protein was undetectable regardless of the cell cycle status in untreated controls (Figures 6c and d). However, upon treatment, p53 as well as its transcriptional target gene p21 was much more greatly elevated in the cycling cells (Figures 6c and d). Immunofluorescence on MEFs treated with Cisplatin also demonstrated much more elevated level of p53 activation and response in the cycling cells (Figure 6e). Thus, the concordant elevation in *p53* expression may provide one possible functional basis for robust p53 responses selectively in the proliferating compartments upon stress.

## Discussion

The regulatory and functional mechanisms of p53 have been under intensive investigations at molecular, cellular and organismal levels to better understand its roles in tumor suppression and stress responses. Aiming to study *p53* expression pattern and regulatory mechanisms both under physiological contexts and in a comprehensive manner, we established an *in vivo* reporter system in BAC transgenic mice that were able to recapitulate endogenous *p53* mRNA expression and identified a highly selective expression pattern for the reporter across diverse tissue compartments in both physiological and pathological conditions. We provided further evidence suggesting that the preferential expression of the reporter in proliferating compartments is critically dependent on both *p53* promoter and 3′UTR elements through transcriptional or posttranscriptional mechanisms. In addition, this study also revealed a distinctive functional difference of p53 in proliferating compartments *versus* their differentiated or quiescent counterparts upon stress.

Previous studies on *p53* mRNA expression and promoter analyses provided a number of relatively separated clues on *p53* expression regulation in general and a few of them were implicated in proliferating cells. However, to our knowledge, there is no commonly recognized theme for *p53* expression pattern *in vivo* and the importance and relevance remained to be further established for many of the *cis*-elements and transacting factors identified in regulating *p53* expression. Here we took a transgenic approach in integrating different lines of studies and shed new insights on *p53* expression and its regulatory mechanisms in a diverse set of conditions *in vivo*. Our results corroborated the existing results and argued strongly for a unified and distinctive expression pattern for *p53 in vivo*.

It becomes increasingly apparent that the studies on the regulatory mechanisms of gene expression can be better oriented and more meaningful with a clear understanding of the expression pattern of the gene. As an immediate early gene, *c-Myc* is induced earlier than *p53* mRNA accumulation^17, 27^ and its mRNA level parallels with *p53* mRNA level in cell lines.^14, 28^ Ectopic c-Myc or N-Myc was able to trans-activate reporters driven by an E-box from the *p53* promoter in 3T3 and neuroblastoma cells.^17, 29^ In an effort to decipher the requirements of *p53* expression *in vivo*, we found the E-box element in the *p53* promoter and its binding by c-Myc contributed significantly to high-level expression of *p53* reporters in the proliferating compartments. Notably, as regulators of cell cycle or proliferation, E2F1,^20^ C/EBP beta,^21^ EGR-1^22^ and Ets-1/2^23^ were also implicated in regulating *p53* expression. In *PMZU* mice with high BAC copy numbers, weak *β*-gal staining was still detected in the proliferating compartments of tissues. In addition, the variations in the degree of reduction of *lacZ* expression in the *PMZU* mice of different ages also suggested a differential dependence on the E-box regulation. Thus, the strength of Myc activity, together with the contribution of other factors may help to coordinate *p53* expression levels with the cellular proliferation state in multiple tissue compartments.

In spite of the dominant *p53* reporter expression pattern identified in the vast majority of tissues examined, there were a few exceptions including the purkinje cells and hepatocytes with detectable levels of *β*-gal staining while being largely quiescent. Interestingly, the E-box mutation also abrogated the reporter expression in liver. Our ChIP analysis suggested that the moderate reporter expression in liver may be regulated by USF-1, which is known to be expressed in liver.^30, 31^ When the hepatocytes reentered the cell cycle, a strong increase in *β*-gal expression suggested that these cells were still subjected to the regulatory mechanisms distinguishing proliferating and quiescent states.

Replacement of *p53* 3′UTR with a commonly used SV40pA greatly reduced *p53* reporter expression in the proliferating compartments, suggesting a general role of the 3′UTR as a positive regulatory elements in supporting *p53* expression. RNA-binding factors such as HuR,^32^ Wig1,^33^ CPEB,^34^ and certain miRNAs were reported to regulate mRNA stability and/or translational efficiency by directly binding on *cis*-elements in *p53* 3′UTR.^25, 26, 35^ Although HuR and Wig1 could stabilize reporter mRNA and/or strengthen its translational efficiency upon DNA damage stress, CPEB did not significantly influence *p53* mRNA level, but instead regulated the *p53* mRNA polyA length and protein level through translation.^34^ In our study, reporter mRNA levels were not significantly altered in the *PZS* mice, thus pointing to the possible translational control in mediating the high-level expression mediated by the 3′UTR sequences.

Our results suggested concerted effects of both transcriptional and posttranscriptional regulations in supporting the selective *p53* expression pattern in proliferating compartments. Known to be anti-proliferative, p53 protein is kept to undetectable levels in normal cells. However, this could also obscure many possible links between p53 expression and its function. For example, proliferative tissues are often more radiosensitive,^36, 37^ and while complex mechanisms may underlie such phenomenon, heightened *p53* basal expression may have a role. Through genetic deletion of *Mdm2* in a *p53* hypomorphic background, we previously discovered that p53 protein stabilization and accumulation only appeared in the proliferating compartments of mice,^6^ a pattern fully recapitulated in the *p53* reporter mice in this study. Here we demonstrated that DNA damage elicited a greater p53 response in the proliferating cells. Therefore, the posttranslational controls seem to act possibly in sequence with the transcriptional and posttranscriptional mechanisms in fine-tuning p53 activity in a variety of cell types depending on their proliferative and differentiation status.

In summary, this study took a genetic approach to address the long existing question of *p53* expression and revealed novel insights suggesting a general intrinsic mechanism for upregulating *p53* in proliferating cells and tissues. Linking to p53 function, the potential advantage and significance for such regulation at the expression level can be several folds: first, higher basal level of p53 would allow a fast and robust protein stabilization/activation and stress response upon stress; on the other hand, a retarded or weak p53 response may protect the cells from apoptosis in the terminally differentiated cells and reduce tissue damage; finally, the switches of cell proliferative state or fate constantly occur for stem cells and progenitors during homeostasis. The coordinated control of *p53* expression may allow the uninterrupted monitoring of proliferation to fulfill its tumor suppressor functions.

As one of the most fundamental cellular processes, cellular proliferation and its regulation are an integral part of development, tissue homeostasis, tissue regeneration and tumorigenesis. Close monitoring of this process by a tumor suppressor controlled at multiple levels may set up a precautionary mode in the cells without causing much disturbance.

## Materials and Methods

### Mice

Mice were bred and maintained under specific pathogen-free conditions and experiments were conducted in accordance with the Institutional Animal Care and Use Committee at the animal facility of Model Animal Research Center of Nanjing University, China. *p53*^*PZU*^ (*PZU*), *p53*^*PMZU*^(*PMZU*) and *p53*^*PZS*^(*PZS*) mice were established on C57BL/6J and CBA mixed background and were backcrossed to C57BL/6J background for three generations. *PZU* mice were crossed to C57BL/6J-*Apc*^Min/+^ mice obtained from Jackson Laboratory (Bar Harbor, ME, USA) to establish *PZU*; *Apc*^Min/+^ mice.

### *In situ* hybridization

Tissues were quickly immersed into cold 4% PFA solution for 1–2 h, dehydrated with 30% sucrose in PBS (pH 7.4) at 4 °C overnight, embedded in OCT and cut into 12 *μ*m slices, then sobbed onto slides covered by APES (amino-propyl-tri-ethoxy-silane) and dehydrated at 50 °C for 2 min and at RT in air for 2 h, and then preserved in −80 °C or treated immediately with a 479 bp biotin-tagged *p53* mRNA sense or antisense probe. Slices were hybridized overnight at 65 °C (70 °C for small intestine) with the *p53* RNA probe; after washed with gradient SSC solution, slices were incubated at 4 °C overnight with rabbit polyclonal antibody tagged with alkaline phosphatase (Roche, Tucson, AZ, USA; 1 : 500) against biotin; signals were developed with NBT/BCIP solution(Roche), and imaged by a bio-microscope (OLYMPUS, Tokyo, Japan). Probes were generated using an *in vitro* transcription kit (Roche). Primers to generate template for probes: 5′-AGTTCATTGGGACCATCCTGG-3′(F), 5′-CGTGCACATAACAGACTTGGC-3′(R).

### Generation of BAC transgenic mice

*p53* BAC (No.bMQ-441J16, Research Genetics, USA) was modified by homologous recombination to insert a *LacZ* reporter gene (from placZattB^38^) together with various alterations of *p53* *cis*-regulatory elements to establish *p53*^*PZU*^, *p53*^*PMZU*^ and *p53*^*PZS*^ reporter constructs. The loxp and loxp511 sequences in the BACe3.6 backbone were then replaced with *Amp* and *Kan* prokaryotic expression cassette. The final BAC constructs were purified by Nucleo Bond^R^ max100 BAC extraction kit (MACHERERY-NAGEL, Düren, Germany), and were verified via sequencing and restriction enzyme mapping. BAC DNA was diluted to 1.0 ng/*μ*l in microinjection buffer, and 1 pl DNA was injected into pronucleus of the fertilized mouse egg from CBA/ C57BL/6J crosses, as previously described.^39^ BAC transgenic copy number was estimated by comparing the photo density of bands representing *p53* reporter transgene to those representing endogenous *p53* allele as identified in Southern blot analysis.

### X-gal staining

Embryos or tissues were fixed with cold 4% PFA for 1.5 h on ice. Embryos were stained in 1 mg/ml X-gal staining solution with gentle shaking for 24 h at 4 °C. Tissues were dehydrated, embedded in OCT and cut into 10*μ*m slices, which were stained in 1 mg/ml X-gal staining solution for 3–10 h at either 25 or 37 °C (for comparing *β*-gal expression levels, staining conditions and time should be consistent between samples). X-gal staining for cells was similar as that for tissues slices except the fixation step: after washed with cold 1 × PBS, cells were fixed with 0.25% glutaraldehyde in 1 × PBS for 5 min as described.^40^

### IHC and immunofluorecence

Tissues were dissected and fixed in cold 4% PFA over night at 4 °C, dehydrated by gradient alcohol (from 50 to 100%), rendered transparent in xylol and embedded in paraffin, then cut in a microtome to slices of 6 *μ*m in thickness and affixed onto the APES coated slides. After deparaffinized and rehydrated, slices were performed with heat-induced epitope retrieval in sodium citrate (pH 6.0), blocked with normal goat serum block solution (Boster, Wuhan, China) for 1 h at room temperature. For IHC, slices were incubated with rabbit polyclonal antibody against *β*-gal (Invitrogen, Shanghai, China; A11131, 1 : 200), rat monoclonal antibody against Ki67 (Dako, Glostrup, Denmark; 1 : 100) or rabbit polyclonal antibody against p53 (Vector, Burlingame, CA, USA; CM5,1 : 500) for 18–22 h at 4 °C. *β*-gal or p53 was detected using Ultra Sensitive S-P (rabbit) kit (MaiXinBio, Fuzhou, China); Ki67 was incubated with the biotin-tagged goat anti-rat secondary antibody (Jackson, West Grove, PA, USA; 1 : 300) for 1 h at RT, then incubated with avidin-tagged horseradish peroxidase. All signals were developed with DAB agents (MaiXinBio) and then stained with hematoxylin. For immunofluorecence analysis, slices were incubated with chicken polyclonal antibody against *β*-gal (Abcam, Cambridge, MA, USA; ab9361, 1 : 200) and rat monoclonal antibody against Ki67 (Dako, 1 : 100) for 18–22 h at 4 °C, and then were incubated with the Cy3-tagged goat anti-chicken (Jackson, 1 : 500), FITC-tagged goat anti-rat antibodies (Jackson, 1 : 500) and the nucleus was stained with Hoechst (Sigma, St. Louis, MO, USA) for an hour at room temperature. Signals were imaged by either a bio- or confocal microscope (OLYMPUS).

### ChIP analysis

For NIH3T3 cells: to make cells in a proliferating state, 2.2 × 10^6^ NIH3T3 cells were cultured in 10-cm dish with 20% fetal bovine serum (FBS) in DMEM for 12 h. To make cells in a quiescent state, 2.2 × 10^6^ NIH3T3 cells were cultured in 10-cm dish with 10% FBS in DMEM until cells reached 80–90% confluency; then cells were cultured with 0.05% FBS in DMEM for next 72 h. Cells were collected, sonicated and ChIP analysis was performed according to manufacturer's instructions (Upstates) with Rabbit polyclonal antibody against c-Myc (Santa Cruz, Santa Cruz, CA, USA; sc-764 ×) or Rabbit IgG (Santa Cruz, Sc2763). Primer sequences used: 5′-CAGCTTTGTGCCAGGAGTCT-3′(F1), 5′-TAACTGTAGTCGCTACCTAC-3′(R1). For embryo and tissues: CHIP was performed as described^41^ with modifications on sonication: sonication for 1 s and pause for 2 s with the total time being 1 min for small intestine, 6 min for liver, and two 3.8 min for embryos. For MEFs: CHIP was performed similarly as 3T3 cells with a few differences: early passage MEFs from *PZU* or *PMZU* mice were plated on 10-cm dish at 1.5 × 10^6^ density and cultured in 0.1% FBS in DMEM for 7–8 h, then cultured with 20% FBS in DMEM for 6 h. 10058-F4 (Sigma) was added to the medium at a final concentration of 180 *μ*M, and MEFS were cultured for another 6 h and was sonicated for 4.8 min with sonication for 1 s, pause for 2 s.

Primers used for embryo, tissues and MEFs: wild type E-box, 5′- ACTTTTCACAAAGCGTTCCT-3′(F2), 5′-TTAGCCAGGGTGAGCACGTG-3′(WTR2); mutant E-box, 5′-ACTTTTCACAAAGCGTTCCT-3′(F2), 5′-TTAGCCAGGGTGAGCACACC-3′(MutantR2).

### RNA and real time PCR

Total RNA samples were isolated using TRIzol (Invitrogen) and were reverse transcribed to cDNA using M-MLV Reverse Transcription kit (Invitrogen) following manufacturer's instructions. Diluted cDNAs were used for Real Time PCR with SYBR Green reagents (Invitrogen) on an ABI Prism Step-One bio-analyzer (Foster City, CA, USA). Sequences of primers are available upon request. Expression data were normalized to *β-actin* mRNA, *18 s rRNA or* endogeneous *p53* mRNA expression. Expression changes were calculated using the ΔΔCt method and expressed as fold change over control. Experiments were repeated three times with similar results.

### Western blotting

MEFs and 3T3 cells were lysed in RIPA buffer supplemented with complete Protease Inhibitor Cocktail Tablets (Roche). The membrane was incubated with anti- p53 (Vector, CM5, 1:200), anti-p21 (Santa Cruz, M19, 1:200), and appropriate secondary antibody (Pierce, Rockford, IL, USA; 1:2000) sequentially. Protein detection was performed using the ECL substrate (Thermo, Rockford, IL, USA) before exposure to film.

### X-ray treatment

Mice were exposed to X-ray irradiation at 6 Gy dosage at a rate of 1.2 Gy per minute using Biological X-ray irradiator (RS2000, Rad Source, Brentwood, TN, USA). Tissues were collected 4 h after X-ray irradiation.

### Statistical analysis

Data are expressed as mean±S.E.M. from triplicates. Statistical analyses were carried out using GraphPad Prism 5 software (GraphPad Software, La Jolla, CA, USA) and *t-*test analysis was performed between two groups. All *P*-values <0.05 were considered statistically significant.

## Abbreviations

*Trp53*: p53 gene or transformation related protein 53 gene
BAC: bacterial artificial chromosome
*c-MYC*: cellular myelocytomatosis oncogene
USF-1: upstream stimulatory factor-1
E-box: enhancer box sequence (CANNTG)
ChIP: chromatin immunoprecipitation
3′UTR: 3′ terminal untranslated region
*LacZ*: E.coli *β*-galactosidase Z gene
EGFP: enhanced green fluorescence protein
SV40pA: simian vacuolating virus 40 polyadenylation sequences
FBS: fetal bovine serum
*Apc*^*min*^: a mutation in the murine *Apc* gene
Brdu: 5-Bromo-2-Deoxy Uridine
