# Supplementary material for: BAC transgenic mice provide evidence that p53 expression is highly regulated in vivo
Source: Cell Death Dis. 2015 Sep 17;6(9):e1878–. doi: 10.1038/cddis.2015.224 (PMC4650433; doi:10.1038/cddis.2015.224)
Supplement: Supplementary Figure Legends [file cddis2015224x1.doc]

**Supplementary Figure 1. c-Myc could bind the conserved E-box in *p53* promoter in proliferating but not quiescent 3T3 cells.**

**Supplementary Figure 2. Preferential *p53* reporter expression in the proliferating compartments in *PZU* mice.**

1. Images of de-skinned *p53* reporter mice with β-Gal staining at P7, P21, 81 day and 13 months of age, respectively. De-skinned mice were whole-mount stained in 1mg/ml X-gal staining solution at 37[°C](https://search.yahoo.com/search;_ylt=A0LEVzPcaZxVQCEAF3NXNyoA;_ylu=X3oDMTE3amtxcHVvBGNvbG8DYmYxBHBvcwM0BHZ0aWQDQjAzNjJfMQRzZWMDcmVsLWJvdA--?p=35+°c&ei=UTF-8&fp=1&fr2=rs-bottom&fr=yfp-t-901) for 10 hours.
2. Upon withdraw of serum in the media, proliferating MEFs gradually moved to a quiescent state. Scale bar=200μm.
3. Immunohistochemistry of Ki67 as an indication of proliferation during liver regeneration after hepatotectomy. Scale bar=200μm.
4. *p53* mRNA expression during liver regeneration after hepatotectomy. Scale bar=200μm.
5. Immunohistochemistry of β-gal and Ki67 in polyps of *PZU*; *Apc*^min^mice. Scale bar=200μm.

**Supplementary Figure 3. *p53* mRNA expression paralleled the cell cycle progression status in MEFs (A) and 3T3 cells (B) stimulated with serum.** *p53* mRNA level was normalized to 18s RNA.

**Supplementary Figure 4. RT-PCR results of *LacZ* mRNA expression as normalized to *p53* mRNA in small intestine, spleen and kidney from *PZU* and *PZS* mice**. *LacZ* mRNA was normalized to endogenous *p53* mRNA.

Values are means ± SEMs. * *P* <0.05; ** *P* <0.01; *** *P* <0.001 (t-test).
