# Supplementary figures and images for: BAC transgenic mice provide evidence that p53 expression is highly regulated in vivo
Source: Cell Death Dis. 2015 Sep 17;6(9):e1878–. doi: 10.1038/cddis.2015.224 (PMC4650433; doi:10.1038/cddis.2015.224)

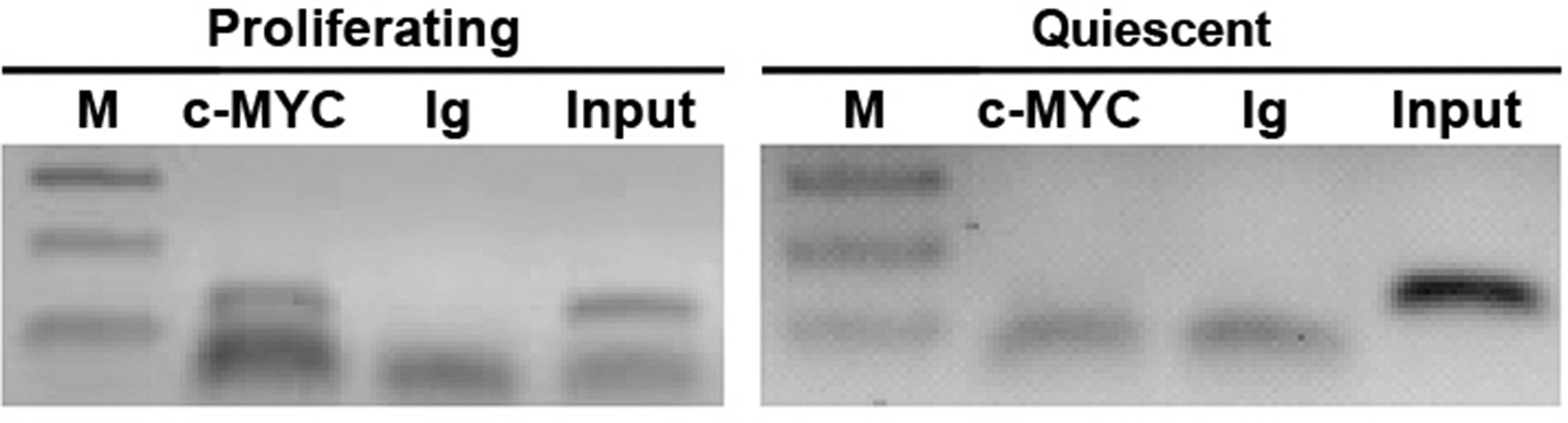

Supplement: Supplementary Figure 1 [file cddis2015224x2.tif]

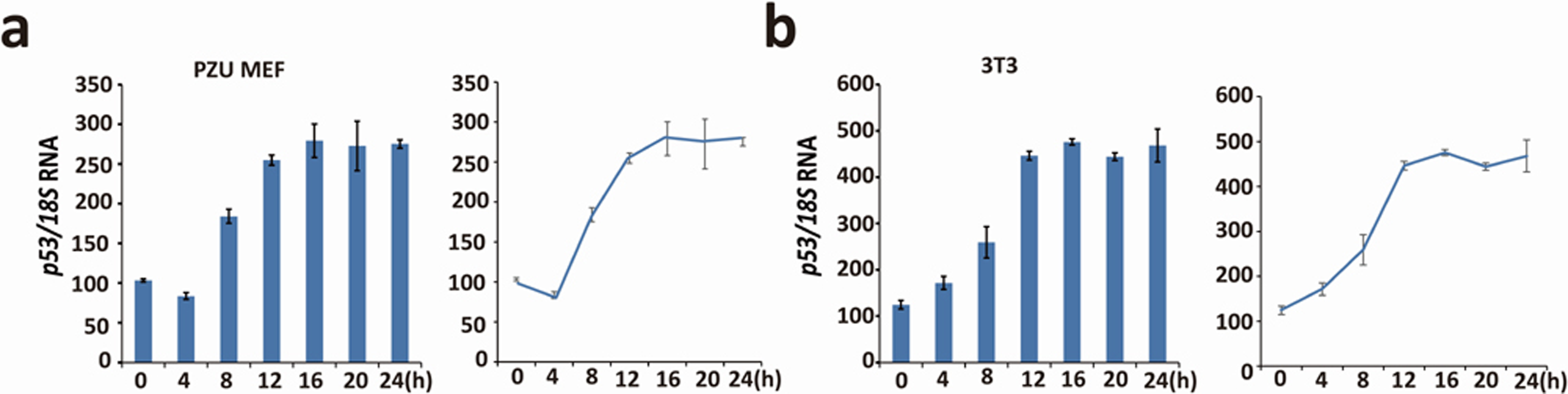

Supplement: Supplementary Figure 3 [file cddis2015224x4.tif]

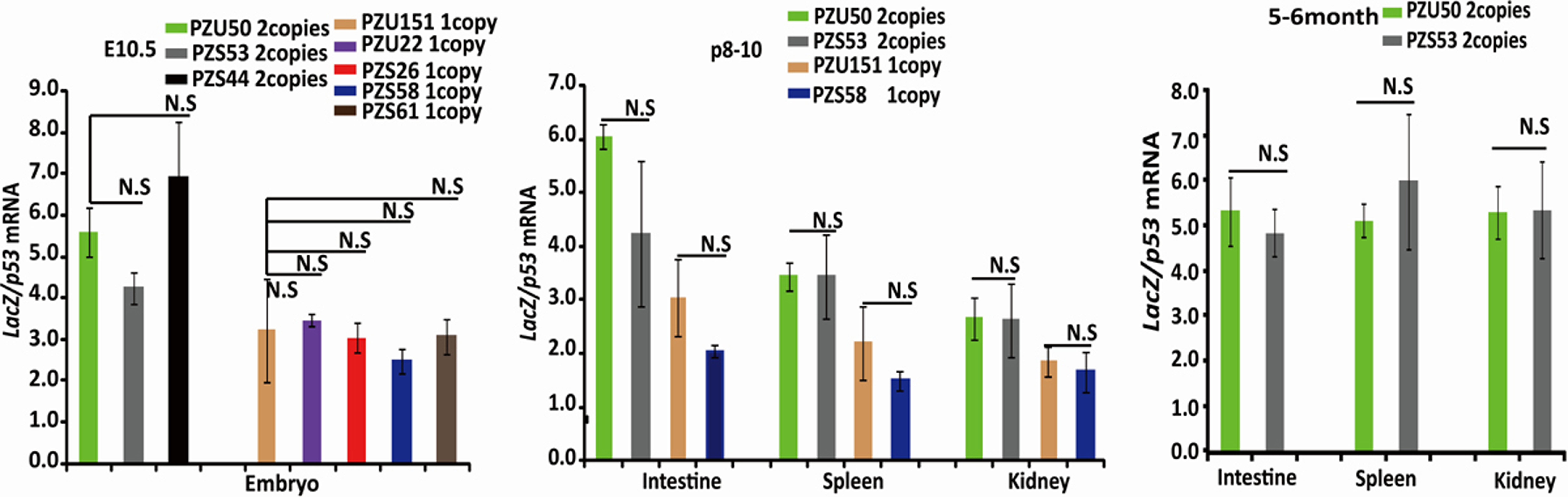

Supplement: Supplementary Figure 4 [file cddis2015224x5.tif]
